# Development of a ^99m^Tc-labeled tetrazine for pretargeted SPECT imaging using an alendronic acid-based bone targeting model

**DOI:** 10.1371/journal.pone.0300466

**Published:** 2024-04-16

**Authors:** Lennart Bohrmann, Christian B. M. Poulie, Cristina Rodríguez-Rodríguez, Stoyan Karagiozov, Katayoun Saatchi, Matthias M. Herth, Urs O. Häfeli

**Affiliations:** 1 Faculty of Pharmaceutical Sciences, University of British Columbia, Vancouver, BC, Canada; 2 Department of Pharmacy, Faculty of Health and Medical Sciences, University of Copenhagen, Universitetsparken, Copenhagen, Denmark; 3 Department of Drug Design and Pharmacology, Faculty of Health and Medicinal Sciences, University of Copenhagen, Universitetsparken, Copenhagen, Denmark; 4 Department of Clinical Physiology, Nuclear Medicine & PET, Rigshospitalet, Blegdamsvej, Copenhagen, Denmark; University of New Hampshire, UNITED STATES

## Abstract

Pretargeting, which is the separation of target accumulation and the administration of a secondary imaging agent into two sequential steps, offers the potential to improve image contrast and reduce radiation burden for nuclear imaging. In recent years, the tetrazine ligation has emerged as a promising approach to facilitate covalent pretargeted imaging due to its unprecedented kinetics and bioorthogonality. Pretargeted bone imaging with TCO-modified alendronic acid (Aln-TCO) is an attractive model that allows the evaluation of tetrazines in healthy animals without the need for complex disease models or targeting regimens. Recent structure-activity relationship studies of tetrazines evaluated important parameters for the design of potent tetrazine-radiotracers for pretargeted imaging. However, limited information is available for ^99m^Tc-labeled tetrazines. In this study, four tetrazines intended for labeling with *fac*-[^99m^Tc(OH_2_)_3_ (CO)_3_]^+^ were synthesized and evaluated using an Aln-TCO mouse model. 3,6-bis(2-pyridyl)-1,2,4,5-Tz without additional linker showed higher pretargeted bone uptake and less background activity compared to the same scaffold with a PEG_8_ linker or 3-phenyl-1,2,4,5-Tz-based compounds. Additionally, improved bone/blood ratios were observed in pretargeted animals compared to animals receiving directly labeled Aln-TCO. The results of this study implicate 3,6-bis(2-pyridyl)-1,2,4,5-Tz as a promising scaffold for potential ^99m^Tc-labeled tetrazines.

## Introduction

In nuclear medicine, the choice of radionuclide for imaging is typically determined by the physical decay properties of the nuclide as it has to match the pharmacokinetic profile of the targeting vector. The optimal choice aims to provide high image contrast and low radiation burden to the patient, two conditions that do not always align [1]. For instance, the slow target accumulation of nanomedicines and antibodies in the order of hours to days requires the use of long-lived nuclides such as ^111^In or ^124^I [2]. On the other hand, short lived positron emission tomography (PET) nuclides such as ^11^C, ^18^F or ^68^Ga decay rapidly and can only be imaged for a few hours before the signal intensity decreases to unusably low levels, which is problematic for slow clearing targeting agents [3].

**Fig 1:**
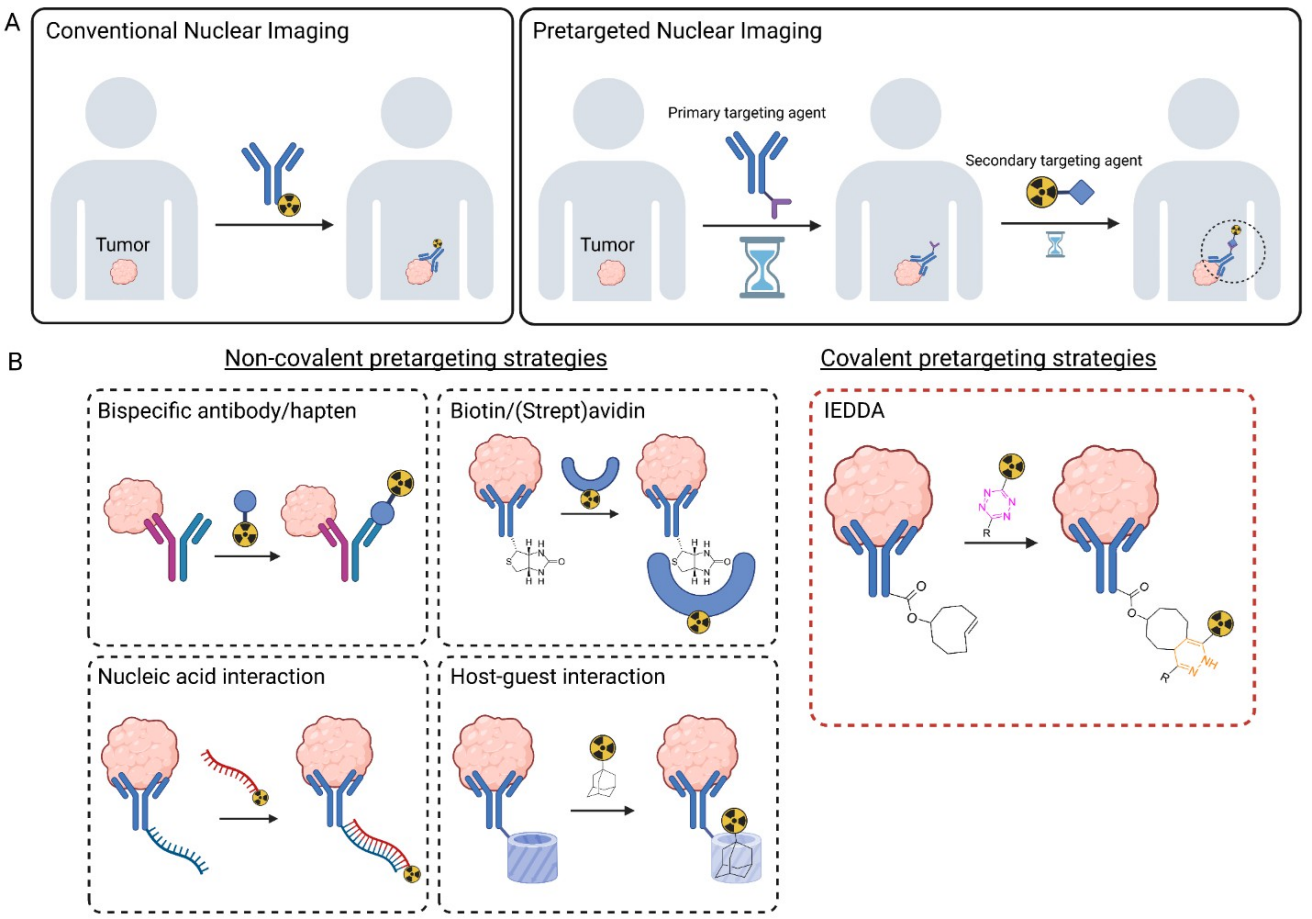
Direct and pretargeted nuclear imaging. (A) In conventional imaging, a targeting vector (i.e., nanomedicine, antibody, aptamer) is labeled with a radionuclide for diagnostic purposes and injected into the patient. Accumulation at the target site can range from days to minutes, depending on the pharmacokinetics of the vector. During this time period, non-binding portions of the vector are eliminated or accumulate in excretory organs such as liver and kidney, thus decreasing image contrast and increasing off-target radiation dose. In the pretargeted setting the pharmacokinetics of the primary targeting vector (slow) and the secondary imaging vector (fast) are decoupled. Non-binding portions of the secondary vector are rapidly excreted, thereby increasing contrast and reducing radiation burden. (B) Selection of pretargeted imaging pairs using non-covalent interactions. The IEDDA reaction between Tzs and TCO is the most promising bioorthogonal click reaction for pretargeted imaging to date.

Pretargeted nuclear imaging offers various advantages over conventional active targeting by decoupling the targeting process from the imaging through the use of a pretargeting pair [4]. This pairing comprises a primary targeting agent and a secondary imaging agent, where the latter binds specifically to the former and is otherwise quickly eliminated. This strategy reduces the radiation dose to non-targeted areas and enhances image contrast (Fig 1) [1, 5–9].

Conceptually, this technique can be distinguished into two categories of pretargeting pairs that interact *via* non-covalent high affinity interactions and covalent bond formations through bioorthogonal click chemistry. The former category uses pretargeting pairs such as (bi)specific antibodies and radiolabeled haptens [10, 11], (strept)avidin-biotin interactions [12], oligonucleotide hybridisation interactions [13] and host-guest interactions such as a the more recently described adamantane/cucurbituril system [14, 15]. The latter category uses click reactions, i.e., chemical reactions that are (among others) characterized by a large thermodynamic driving force, high yielding and only generating innocuous by-products [16]. Furthermore, since the goal for pretargeted nuclear medicine is *in vivo* use, these reactions must be bioorthogonal, meaning they are selective enough not to interfere with physiological processes and proceed kinetically fast under physiological constraints of pH and temperature [17].

With regards to these requirements, arguably the most promising bioorthogonal click to date is the tetrazine (Tz) ligation. Mechanistically, the Tz ligation is a two-step process that involves an enthalpy driven inverse electron-demand Diels-Alder (IEDDA) [4 + 2] cycloaddition between an electron-deficient Tz and an electron-rich dienophile, such as an alkyne or alkene, most often *trans*-cyclooctene (TCO). This step is followed by an entropy driven retro Diels-Alder reaction, where a stable dihydropyridazine or pyridazine cycloadduct is formed under the elimination of N_2_ [18, 19]. Due to its unprecedented reaction rate of up to 10^7^ M^-1^s^-1^ [20], the Tz ligation has amassed considerable interest for pretargeted imaging *in vivo*. After the first *in vivo* study using a TCO functionalized CC49 antibody and an ^111^In labeled Tz in 2010 [21], numerous studies investigated the IEDDA reaction for the purpose of pretargeted nuclear medicine with diagnostic and therapeutic radionuclides [1, 6, 22–25].

Although ^99m^Tc-labeled Tzs remain relatively rare in the literature, there are advantages to this radionuclide that warrant further evaluation. Firstly, ^99m^Tc is readily available from generators and does not require extensive infrastructure and specialized instruments such as cyclotrons for the production of PET isotopes. Secondly, due to rhenium being a third-row congener of technetium, any promising ^99m^Tc-labeled Tz could theoretically be used for pretargeted theranostic applications using ^188^Re [26]. While many ^99m^Tc radiolabeling techniques involve one-pot processes wherein Tc(VII) undergoes reduction in a kit, transforming into either Tc(V) or Tc(IV) and subsequently forming a complex with a chelator. However, there remains the potential for technetium to interact with various N or O atoms within the chelating compound, leading to uncontrolled redox processes or the formation of technetium oxides. The use of the Tc(I) tricarbonyl precursor mitigates these issues, facilitating the exchange of the three water molecules with a suitable chelator. This precursor is highly compact, possessing the smallest size among all technetium compounds, and maintains exceptional stability in its Tc(I) state [27–29].

The aim of the current study was to evaluate the performance of four ^99m^Tc-labeled Tzs using an alendronic acid based pretargeting agent functionalized with a TCO moiety (Aln-TCO). This bisphosphonate has been used previously in mice [30–33], rats [34] and dogs [35], and is suitable for testing the *in vivo* click performance of Tzs due to its rapid accumulation at sites of active bone remodeling, followed by fast renal excretion [36, 37]. Pretargeted imaging is thus possible without long intervals between administration of primary and secondary agents and does not require complex tumor models or antibody-based targeting agents that add further layers of complexity to the system [38–40].

While fast reaction kinetics are an absolute prerequisite to successful in *vivo click* reactions, it is not surprising that the physicochemical properties of the Tzs affect their pharmacokinetic profile and play a significant role in their performance. Linker chemistry, charge of the radiometal complex and overall lipophilicity of the Tz have a substantial impact on the *in vivo* performance of structurally related Tzs [32, 41–44]. To find the optimal Tz, we evaluated two Tzs with and without PEG_8_ linker and a novel tricarbonyl based chelator for their effectiveness of *in vivo* pretargeted imaging.

## Materials and methods

### General

Chemicals were purchased from Sigma-Aldrich (St. Louis, MO, USA), Merck (Darmstadt, Germany) or VWR (Radnor, PA, USA), unless otherwise specified. ^1^H and ^13^C NMR spectra were recorded on a Bruker Ascend-400 magnet (Billerica, MA, USA) at 400.13 MHz or a 600 MHz Bruker Avance III HD and analyzed using MNova software (Mestrelab Research; Santiago de Compostela, Spain). Graphing and statistical analysis was performed using Origin 2019 software (OriginLab; Northampton, MA, USA) and multi-panel figures were prepared using Adobe Illustrator CC (San Jose, CA, USA). Data are represented as mean ± SD, n = 3, unless otherwise noted. Statistical significance (*, p < 0.05) was determined using two-way repeated measurement ANOVA. The distribution coefficient at physiological pH (logD_7.4_) was calculated using the software chemicalize (chemicalize.com; ChemAxon, Budapest, Hunagry). ClogD values were calculated for the tetrazines 1–4 without the metal complex.

Preparative HPLC for Aln-TCO was carried out on an Ultimate Thermo SCIENTIFIC HPLC system with an LPG-3200BX pump, a Rheodyne 9721i injector, a 10 mL loop, an MWD-3000SD detector (200, 210, 225 and 254 nm), and a Gemini-NX C18 (250 × 21.2 mm, 5 μm) column for preparative purifications. Solvent A: H_2_O + 0.1% TFA; Solvent B: MeCN-H_2_O 9:1 + 0.1% TFA. For HPLC control, data collection and data handling, Chromeleon software v. 6.80 was used. UPLC-MS spectra were recorded using an Acquity UPLC H-Class Waters series solvent delivery system equipped with an autoinjector coupled to an Acquity QDa and TUV detectors installed with an Acquity UPLC®BEH C18 (50 × 2.1 mm, 1.7 μm) column. Solvent A: 5% aq MeCN + 0.1% HCO_2_H: Solvent B: MeCN + 0.1% HCO_2_H. Usually, gradients from A:B 1:0 to 1:1 (5 min) or A:B 1:0 to 0–50 (5 min), were performed depending on the polarity of the compounds. For data collection and data handling, MassLynx software was used. Preparative HPLC for tetrazines was carried out on an Agilent 1200 series instrument equipped with a Phenomenex Luna C18 (250 x 10 mm, 10 μm) column operated at a flow of 4 mL/min. Solvent A: H_2_O + 0.1% TFA; Solvent B: MeCN-H_2_O 9:1 + 0.1% TFA. Gradient: 0–3 min: 95% solvent A; 3–20 min: ramp to 100% solvent B; 20–23 min: 100% solvent B.

### Chemical synthesis

(E)-(4-(((cyclooct-4-en-1-yloxy)carbonyl)amino)-1-hydroxybutane-1,1-diyl)bis(phosphonic acid) (**Aln-TCO**)

Equatorial TCO-PNB ester[45] (29 mg, 0.11 mmol) was dissolved in DMF (1 mL) and added dropwise to a premixed solution of sodium alendronate trihydrate (32 mg, 0.1 mmol) and Et_3_N (181 μL, 1.3 mmol) in H_2_O (1 mL), and the mixture stirred overnight in the dark. The reaction mixture was diluted to 7 mL with H_2_O (containing 0.5% TFA) and was submitted to preparative HPLC. All fraction containing pure compound were lyophilized, producing a white solid (4.2 mg 12%). ^1^H NMR (600 MHz, D_2_O) δ 5.79–5.64 (m, 2H), 4.67 (bs, 1H), 3.15 (t, *J* = 6.4 Hz, 2H), 2.41–2.33 (m, 1H), 2.20–2.14 (m, 1H), 2.10 (dtd, *J* = 14.2, 7.0, 4.3 Hz, 1H), 2.06–1.94 (m, 3H), 1.94–1.85 (m, 2H), 1.85–1.77 (m, 3H), 1.77–1.70 (m, 1H), 1.70–1.60 (m, 2H), 1.60–1.46 (m, 1H) ppm. ^13^C NMR (151 MHz, D_2_O) δ 158.5, 130.3, 130.0, 77.2, 73.1 (t, *J*_C-P_ = 143.4 Hz), 40.7, 33.4, 33.2, 30.8, 25.1, 24.4, 23.8 (t, *J*_C-P_ = 6.1 Hz), 21.6 ppm. ^31^P NMR (162 MHz, D_2_O) δ 19.34 ppm.

#### Synthesis of tetrazines

Synthesis of tetrazines for this work was based on a strategy to react NHS-ester precursors with amine-modified building blocks to afford tetrazines with or without PEG_8_ linker. Synthesis of 3,6-di-2-pyridil substituted Tzs **1** and **3** started from *2*,*5-dioxocyclopentyl 5-oxo-5-((6-(6-(pyridin-2-yl)-1*,*2*,*4*,*5-tetrazin-3-yl)pyridin-3-yl)amino)pentanoate* (B3), while the synthesis of 3-phenyl substituted Tzs **2** and **4** started from *2*,*5-dioxopyrrolidin-1-yl 5-((4-(1*,*2*,*4*,*5-tetrazin-3-yl)phenyl)amino)-5-oxopentanoate* (H3). Synthesis of the Tz scaffolds followed previously reported procedures, [46, 47] and NHS-functionalization was adapted from Selvaraj *et al*. [48]. A detailed description about chemical synthesis of Tz precursors and the chelator is provided in the supplementary information (S1 File).6-(((carboxymethyl)(2-(5-oxo-5-((6-(6-(pyridin-2-yl)-1,2,4,5-tetrazin-3-yl)pyridin-3-yl)amino)pentanamido)ethyl)amino)methyl)picolinic acid (**1**)

B3 (20.5 mg, 0.056 mmol) and NH_2_-chelator (7.1 mg, 0.028 mmol) were added to a dry vial under nitrogen atmosphere. To the vial was added 2.5 mL anhydrous DMF and *N*,*N*-diisopropylethylamine (9.8 μL, 0.056 mmol). The vial was stirred at RT for 12 h, before the solvent was removed under reduced pressure and the title compound was afforded by preparative HPLC as a purple solid (6.45 mg, 38%). ^1^H NMR (400 MHz, MeOH-d4) δ 8.99 (d, *J* = 2.0 Hz, 1H), 8.86 (d, *J* = 4.4 Hz, 1H), 8.72 (dd, *J* = 26.2, 8.3 Hz, 1H), 8.40 (dd, *J* = 8.7, 2.4 Hz, 1H), 8.19–8.11 (m, 1H), 8.06 (t, *J* = 7.7 Hz, 1H), 7.72 (t, *J* = 6.8 Hz, 1H), 4.79 (s, 1H), 4.23 (d, *J* = 9.1 Hz, 1H), 3.58 (dt, *J* = 9.8, 9.2 Hz, 2H), 2.52 (t, *J* = 7.2 Hz, 1H), 2.39 (t, *J* = 7.2 Hz, 1H), 2.01 (p, *J* = 7.2 Hz, 1H) ppm.

29,33-dioxo-33-((6-(6-(pyridin-2-yl)-1,2,4,5-tetrazin-3-yl)pyridin-3-yl)amino)-4,7,10,13,16,19,22,25-octaoxa-28-azatritriacontanoic acid (**B4**)

To a dry microwave vial were added NH2-PEG_8_-propionate (71 mg, 0.16 mmol) and B3 (159 mg, 0.34 mmol). 5 mL anhydrous DMF were added, and the vial was heated to 130°C for 1.5 h. After removal of DMF under reduced pressure, the title compound was obtained by preparative HPLC as a purple resin (69 mg, 0.01 mmol, 61%). ^1^H NMR (400 MHz, DMSO-d6): δ 12.12 (s, 1H), 10.53 (s, 1H), 9.05 (d, J = 2.5 Hz, 1H), 8.93 (dt, J = 4.7, 1.3 Hz, 1H), 8.67–8.52 (m, 2H), 8.43 (dd, J = 8.7, 2.5 Hz, 1H), 8.15 (td, J = 7.8, 1.8 Hz, 1H), 7.89 (t, J = 5.6 Hz, 1H), 7.72 (ddd, J = 7.7, 4.7, 1.2 Hz, 1H), 3.59 (t, J = 6.4 Hz, 3H), 3.55–3.45 (m, 28H), 3.42 (t, J = 5.9 Hz, 2H), 3.21 (q, J = 5.8 Hz, 2H), 2.44 (td, J = 6.9, 6.4, 3.4 Hz, 4H), 2.18 (t, J = 7.3 Hz, 2H), 1.86 (p, J = 7.4 Hz, 2H) ppm. ^13^C NMR (400 MHz, DMSO-d6): δ 173.07, 172.58, 172.16, 163.53, 151.07, 150.68, 141.75, 138.26, 127.04, 126.58, 125.36, 124.66, 70.24, 70.16, 70.09, 70.05, 69.63, 66.70, 38.97, 36.15, 35.21, 34.90, 21.40 ppm.

2,5-dioxopyrrolidin-1-yl 29,33-dioxo-33-((6-(6-(pyridin-2-yl)-1,2,4,5-tetrazin-3-yl)pyridin-3-yl)amino)-4,7,10,13,16,19,22,25-octaoxa-28-azatritriacontanoate (**B5**)

B4 (55 mg, 0.07 mmol) was added to a dry microwave vial and dissolved in 2 mL anhydrous DCM. *N*,*N*-disuccinimidyl carbonate (25 mg, 0.1 mmol) was added and the solution was cooled to 0°C. 5 μL pyridine and 83 μL triethylamine were added and the solution was stirred at 0°C for another 10 minutes before the ice bath was removed and the solution was allowed to warm up to room temperature. After 2 h, the solvent was evaporated, and the crude product was redissolved in 20 mL DCM. The organic phase was washed with water (3 times, 20 mL each) and 20 mL brine before it was dried over sodium sulfate and filtered. The solvent was removed under reduced pressure to yield the product as a purple flakey solid (51 mg, 0.06 mmol, 83%). ^1^H NMR (400 MHz, DMSO-d6): δ 10.54 (s, 1H), 9.05 (d, J = 2.5 Hz, 1H), 8.93 (dt, J = 4.7, 1.3 Hz, 1H), 8.60 (dd, J = 11.5, 8.3 Hz, 2H), 8.43 (dd, J = 8.7, 2.5 Hz, 1H), 8.15 (td, J = 7.8, 1.8 Hz, 1H), 7.89 (t, J = 5.6 Hz, 1H), 7.72 (ddd, J = 7.6, 4.7, 1.2 Hz, 1H), 3.71 (t, J = 6.0 Hz, 2H), 3.51 (d, J = 5.4 Hz, 28H), 3.42 (t, J = 5.9 Hz, 2H), 3.21 (q, J = 5.8 Hz, 2H), 2.92 (t, J = 6.0 Hz, 2H), 2.81 (s, 4H), 2.44 (t, J = 7.4 Hz, 2H), 2.18 (t, J = 7.3 Hz, 2H), 1.86 (p, J = 7.4 Hz, 2H) ppm. ^13^C NMR (400 MHz, DMSO-d6) δ 172.16, 170.57, 163.25, 151.07, 150.68, 141.75, 139.00, 138.26, 127.04, 126.58, 125.36, 124.66, 70.24, 70.14, 70.05, 69.63, 65.69, 38.97, 36.15, 34.90, 32.07, 25.91, 21.40 ppm.

6-(2-(carboxymethyl)-6,34,38-trioxo-38-((6-(6-(pyridin-2-yl)-1,2,4,5-tetrazin-3-yl)pyridin-3-yl)amino)-9,12,15,18,21,24,27,30-octaoxa-2,5,33-triazaoctatriacontyl)picolinic acid (**3**)

B5 (25 mg, 0.028 mmol) and NH_2_-chelator (6 mg, 0.024 mmol) were added to a dry vial under nitrogen atmosphere. To the vial was added 2 mL anhydrous DMF and *N*,*N*-diisopropylethylamine (11 μL, 0.66 mmol). The vial was stirred at RT for 12 h, before the DMF was removed under reduced pressure and the title compound was afforded by preparative HPLC as a purple resin (7 mg, 28%). ^1^H NMR (400 MHz, MeOH-d4) δ 8.94 (d, *J* = 2.1 Hz, 1H), 8.77 (d, *J* = 4.6 Hz, 1H), 8.65 (t, *J* = 8.8 Hz, 2H), 8.36 (dd, *J* = 8.7, 2.4 Hz, 1H), 8.11–8.03 (m, 2H), 7.99 (t, *J* = 7.8 Hz, 1H), 7.63 (dd, *J* = 10.2, 5.6 Hz, 2H), 4.68 (s, 2H), 4.13 (d, *J* = 8.5 Hz, 2H), 3.60 (t, *J* = 5.9 Hz, 2H), 3.55–3.46 (m, 32H), 3.27 (t, *J* = 5.2 Hz, 2H), 3.20 (s, 7H), 2.46–2.37 (m, 4H), 2.22 (t, *J* = 7.3 Hz, 2H), 1.96–1.87 (m, 2H) ppm. ^13^C NMR (101 MHz, MeOD) δ 175.75, 175.43, 174.34, 169.05, 164.65, 164.49, 152.64, 151.48, 148.92, 145.24, 142.67, 140.80, 140.40, 139.65, 126.26, 125.63, 70.55, 67.95, 59.34, 57.01, 55.98, 49.00, 40.35, 37.29, 36.86, 36.29, 36.00 ppm.

6-(((2-(5-((4-(1,2,4,5-tetrazin-3-yl)phenyl)amino)-5-oxopentanamido)ethyl)(carboxymethyl)amino)methyl)picolinic acid (**2**)

H3 (13.9 mg, 0.045 mmol) and the NH_2_-chelator (7.1 mg, 0.028 mmol) were added to a dried vial under nitrogen atmosphere. To the vial was added 2 mL anhydrous DMF and DIPEA (19 μL, 0.114 mmol) were added. The reaction was for 16 h at RT, before the solvent was evaporated and the product was purified using preparative HPLC chromatography to afford the title compound as a pink solid (6.5 mg, 44%). ^1^H NMR (400 MHz, MeOH-d4) δ 10.27 (s, 1H), 8.47 (d, J = 8.7 Hz, 2H), 8.06 (dd, J = 12.1, 7.5 Hz, 2H), 7.83 (d, J = 8.7 Hz, 2H), 7.74 (d, J = 7.3 Hz, 1H), 4.60 (s, 2H), 3.84 (s, 2H), 3.57 (d, J = 5.0 Hz, 2H), 3.35 (s, 2H), 2.50 (t, J = 7.1 Hz, 2H), 2.40 (t, J = 6.9 Hz, 2H), 2.07–1.98 (m, 2H) ppm. ^13^C NMR (400 MHz, MeOH-d4) δ 176.63 (s), 174.30 (s), 167.31 (s), 158.97 (s), 144.61 (s), 140.62 (s), 129.93 (s), 128.09 (d, J = 19.4 Hz), 125.77 (s), 120.97 (s), 59.31 (s), 56.27 (s), 37.00 (s), 36.61 (s), 35.47 (s), 22.39 (s).

33-((4-(1,2,4,5-tetrazin-3-yl)phenyl)amino)-29,33-dioxo-4,7,10,13,16,19,22,25-octaoxa-28-azatritriacontanoic acid (**H4**)

H3 (42 mg, 0.11 mmol) and NH2-PEG_8_-propionate (41mg, 0.09 mmol) were added to a dried micro-wave vial under nitrogen atmosphere. 2 mL anhydrous DMF and 16 μL (0.11 mmol) triethylamine were added and the mixture was stirred at RT for 18 h. The title compound was obtained by preparative HPLC as a red oil. (65 mg, 82%). ^1^H NMR (400 MHz, DMSO-d6): δ 10.52 (s, 1H), 10.30 (s, 1H), 8.51–8.43 (m, 2H), 7.94–7.85 (m, 3H), 3.60 (t, J = 6.4 Hz, 3H), 3.51 (dd, J = 2.6, 1.7 Hz, 28H), 3.42 (t, J = 5.9 Hz, 2H), 3.21 (q, J = 5.8 Hz, 2H), 2.48–2.36 (m, 4H), 2.17 (t, J = 7.4 Hz, 2H), 2.08 (s, 4H), 1.84 (p, J = 7.4 Hz, 2H) ppm. ^13^C NMR (400 MHz, DMSO-d6): δ 172.58, 171.69, 171.58, 165.08, 157.75, 143.56, 128.68, 125.84, 119.25, 118.03, 69.75, 69.70, 69.67, 69.60, 69.56, 69.13, 66.20, 38.47, 35.80, 34.71, 34.46, 21.02 ppm.

2,5-dioxopyrrolidin-1-yl 33-((4-(1,2,4,5-tetrazin-3-yl)phenyl)amino)-29,33-dioxo-4,7,10,13,16,19,22,25-octaoxa-28-azatritriacontanoate (**H5**)

Synthesis of the title compound was performed analogous to B5, affording a pink resin in 85% yield. ^1^H NMR (400 MHz, DMSO-d6): δ 10.51 (s, 1H), 10.29 (s, 1H), 8.50–8.42 (m, 2H), 7.93–7.84 (m, 3H), 5.75 (s, 1H), 3.71 (t, J = 6.0 Hz, 2H), 3.57–3.47 (m, 28H), 3.41 (t, J = 5.9 Hz, 2H), 3.20 (q, J = 5.8 Hz, 2H), 2.92 (t, J = 6.0 Hz, 2H), 2.81 (s, 4H), 2.39 (t, J = 7.4 Hz, 2H), 2.16 (t, J = 7.4 Hz, 2H), 1.83 (p, J = 7.5 Hz, 2H) ppm. ^13^C NMR (400 MHz, DMSO-d6): δ 172.17, 172.07, 170.57, 167.79, 165.57, 158.24, 144.05, 129.16, 126.33, 119.74, 70.24, 70.19, 70.14, 70.04, 69.62, 65.69, 55.38, 38.96, 36.29, 34.95, 32.07, 25.90, 21.51 ppm.

6-(35-((4-(1,2,4,5-tetrazin-3-yl)phenyl)amino)-2-(carboxymethyl)-3,31,35-trioxo-6,9,12,15,18,21,24,27-octaoxa-2,30-diazapentatriacontyl)picolinic acid (**4**)

H5 (57 mg, 0.071 mmol) and NH_2_-chelator (15 mg, 0.06 mmol) were added to a dry vial. The vial was capped, evacuated and backfilled with nitrogen three times. 2 mL dry DMF and *N*,*N*-diisopropylethylamine (20.6 μL, 0.12 mmol) were added. The vial was stirred at room temperature for 12 h, before the title compound was obtained by preparative HPLC as a red oil (5 mg, 9%). ^1^H NMR (600 MHz, MeOH-d4): δ 10.27 (s, 1H), 8.56–8.50 (m, 2H), 8.22–8.17 (m, 1H), 8.14–8.06 (m, 1H), 7.89–7.79 (m, 2H), 7.77–7.72 (m, 1H), 4.82 (s, 4H), 4.30 (s, 2H), 3.72 (t, J = 6.0 Hz, 2H), 3.66 (t, J = 5.7 Hz, 2H), 3.62–3.57 (m, 28H), 3.57–3.53 (m, 4H), 3.38 (q, J = 5.1 Hz, 2H), 2.55–2.44 (m, 4H), 2.32 (t, J = 7.4 Hz, 2H), 2.01 (p, J = 7.4 Hz, 2H) ppm. ^13^C NMR (600 MHz, MeOH-d4): δ 174.46, 174.07, 172.65, 167.24, 165.91, 157.58, 150.79, 147.55, 143.21, 139.44, 128.63, 127.67, 127.09, 126.90, 124.97, 119.64, 70.05, 70.03, 69.99, 69.90, 69.15, 66.53, 57.92, 55.77, 54.37, 38.95, 35.88, 35.65, 34.80, 34.68, 21.39 ppm.

### Radiolabeling

^99m^TcO4^-^ was converted to *fac*-[^99m^Tc(OH_2_)_3_ (CO)_3_]^+^ according to the manufacturer’s instructions. Briefly, ca. 740 MBq of generator derived ^99m^TcO4^-^ in saline (0.6–1 mL) was added to the CRS kit for tricarbonyl and heated in a boiling water bath for 30 minutes. The vial was allowed to cool to room temperature and neutralized to a pH of approximately 7 using 1M HCl. Conversion of the kit was monitored using instant-thin layer chromatography (ITLC) strips from Biodex (Cat# 150–005, Shirley, NY, USA) and 1% HCl in MeOH as mobile phase. The activity of radioactive samples was measured using a CRC-55tR dose calibrator (Capintec; Florham Park, NJ, USA). For labeling of Tzs, an aliquot of the neutralized *fac*-[^99m^Tc(OH_2_)_3_ (CO)_3_]^+^ solution was mixed with the tetrazines (1 mg, dissolved in 9:1 water to MeCN, 50 μL) and put on a shaker at 70°C for 30 min, 700 rpm. For purification, Tzs were loaded onto chromafix C18 SPE cartridges (Macherey-Nagel; Düren, Germany), washed with 3 mL water and eluted in several fractions using 0.25 mL aliquots of 25% EtOH. A Biotage V10 solvent evaporator (Uppsala, Sweden) was used to remove the EtOH and the tetrazines were redissolved in saline for *in vivo* administration. Radiochemical purity was assessed using radio ITLC or analytical radio-HPLC chromatography on a Waters (Milford, MA, USA) Alliance e2695 separations module coupled to a Waters 2489 UV/VIS detector and a Scan-RAM radio-TLC and HPLC detector (LabLogic, Sheffield, UK) installed with a Waters Atlantis T3 column (C18, 4.6 x 150 mm) and a Waters Atlantis T3 Sentry Guard Cartridge (C18, 2.1 x 10 mm). Solvent A: H_2_O + 0.1% TFA; Solvent B: MeCN-H_2_O 9:1 + 0.1% TFA. Gradient: 0–3 min: 95% solvent A; 3–23 min: ramp to 100% solvent B; 23–25 min: 100% solvent B; flow: 1 mL/min.

### SPECT/CT imaging and biodistributions

The study was conducted in compliance with the guidelines set by the Canadian Council on Animal Care (CCAC) and approved by the Animal Care Committee (ACC) at the University of British Columbia (A20-0132). Healthy Balb/c female mice (~25 g) were anesthetized using isoflurane delivered via a precision vaporizer (5% in oxygen for induction, between 1.5 and 2.5% in oxygen for maintenance) and received 100 μL of a 2 mg/kg solution of Aln-TCO in saline (pretargeted groups), or 100 μL saline (control groups) *via* intravenous injection. After 1 h, mice were anesthetized again and received another i.v. injection containing 100 μL of ^99m^Tc labeled Tzs in saline, with an average activity of 17.58 MBq, as well as a subcutaneous injection of Lactated Ringer’s solution (0.5 mL) to ensure hydration before each imaging scan. After the injection, a VECTor/CT multimodal preclinical scanner (MILabs, The Netherlands) equipped with a HEUHR-1 mm mouse pinhole collimator was used to obtain static whole-body images. The first scans were obtained with a single frame lasting 25 minutes, followed by scans at 2- and 6-hours post-injection, each with a single frame lasting 40 and 50 minutes, respectively.

Throughout the scanning process, mice were maintained under isoflurane anesthesia and kept warm with a heating pad to ensure constant body temperature. Following each SPECT acquisition, a whole-body CT scan was performed to obtain anatomical information and the two images were registered. The ^99m^Tc photopeak window was centered at 140 keV with a 25% energy window width. SPECT image reconstructions were carried out using a pixel-ordered subset expectation maximization (POSEM) algorithm with 16 subsets, 6 iterations, and an isotropic 0.4 mm voxel grid to enable quantitative analysis. The images were decay corrected, and after CT registration, attenuation correction was applied. For visual representation, the reconstructed volumes of SPECT scans were post-filtered with a 3D Gaussian filter. CT scans were conducted using a tube setting of 55 kV and 615 μA, and 2 frames of 180 projections over 360 degrees were captured in step and shoot rotation mode. The acquired projection data was reconstructed using SkyScan NRecon software to generate a 3D CT image on 0.169 mm^3^ voxel size. Volumes of interest (VOIs), in the left ventricle of the heart (blood pool), liver (n = 3), bladder, kidney (n = 2), shoulder and knee joint (n = 2, respectively) were manually defined using AMIDE [49] (v.1.0.5) to determine the time activity pattern per target organ. The average organ activity per volume was obtained from the SPECT images and the %ID/g of tissue were extracted from each organ. To relate the scanner units (counts/pixel) to radioactivity concentration (MBq/mL), a calibration factor was determined scanning a source with a known concentration of ^99m^Tc. Following the last time imaging time point (6 h, except mice injected with ^99m^Tc-**3**, which were sacrificed 24 h p.i.), mice were sacrificed via CO_2_ asphyxiation under isoflurane anesthesia. Blood was recovered via cardiac puncture and organs of interest were dissected out, cleaned and weighed. The bone uptake of the shoulder was measured by measuring the activity of scapula and humerus, cut below the elbow joint, while knee uptake was measured in the femur, cut below the knee joint. Bone samples were thoroughly cleaned from muscle and connective tissue. The activity was quantified on a calibrated Cobra II Autogamma counter (Packard Instruments, USA) and decay corrected to the time of injection.

## Results and discussion

### *In vivo* pretargeting of ^99m^Tc-labeled tetrazines

**Fig 2:**
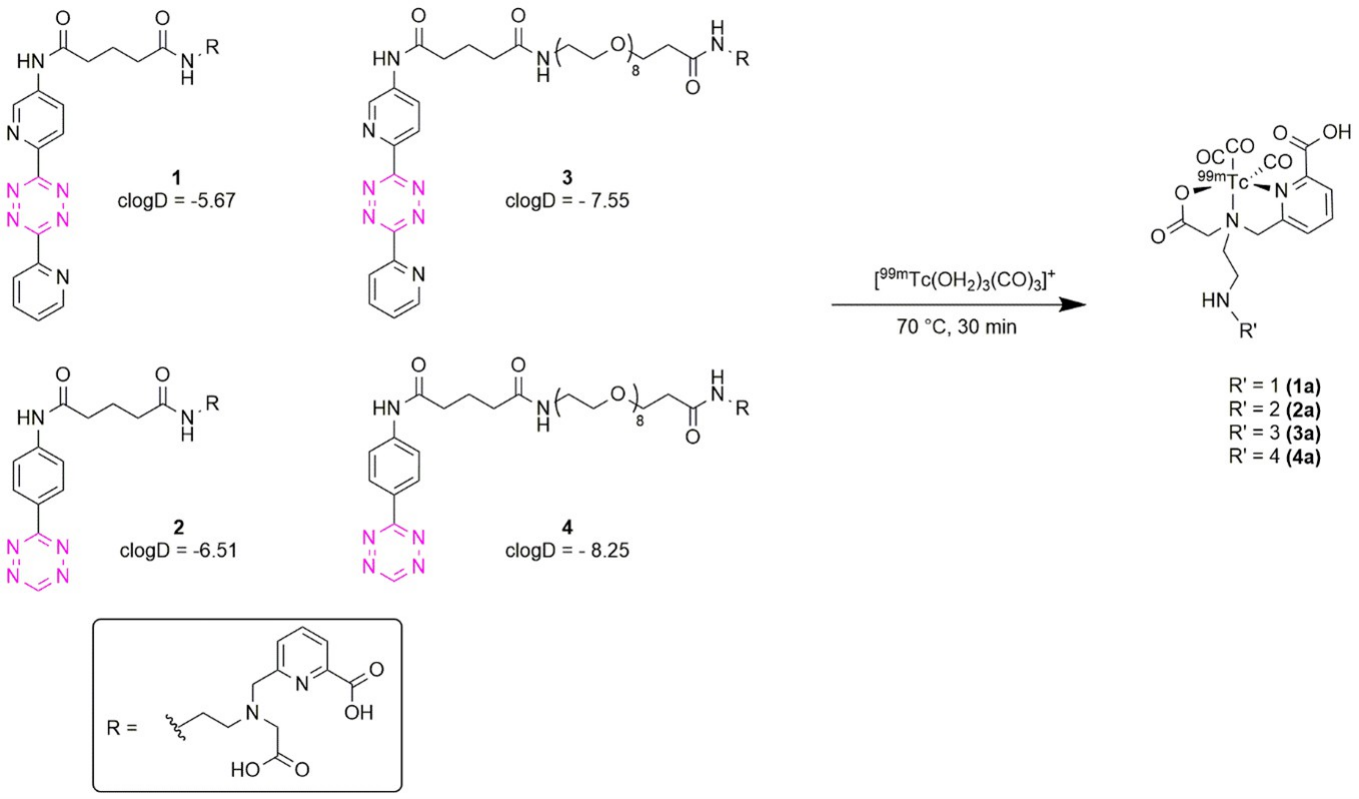
Tetrazines for labeling with ^99m^Tc. The two Tz scaffolds have previously been evaluated and demonstrated good stability and fast kinetics, permitting in vivo pretargeted imaging and therapy with other isotopes. Tetrazines 1 and 2 are directly linked to the chelator, while Tzs 3 and 4 contain a longer PEG_8_ spacer, which separates the tetrazine moiety from the chelator.

A total of four Tzs were synthesized for ^99m^Tc-labeling (Fig 2). The Tzs are based on two well described scaffolds, namely 3-phenyl-1,2,4,5-Tz that is characterized by fast kinetics due to minimal steric hindrance and 3,6-bis(2-pyridyl)-1,2,4,5-Tz that achieves fast kinetics by virtue of the electron withdrawing effect of the pyridines [1, 50]. Both of these Tz scaffolds have previously been evaluated for *in vivo* pretargeted imaging and therapy with a plethora of isotopes such as ^11^C [33, 51], ^18^F [42, 52, 53], ^68^Ga [41, 54], ^111^In [21, 40], ^64^Cu [43, 55], ^177^Lu [6, 56], ^44^Sc [34], ^212^Pb [57] and ^225^Ac [58, 59]. To allow radiolabeling of the tetrazines with, a chelator that is able to coordinate ^99m^Tc from the organometallic precursor *fac*-[^99m^Tc(OH_2_)_3_ (CO)_3_]^+^ [60] was either directly attached to the Tz scaffold (**1**, **2**) or *via* a longer PEG_8_-linker to reduce potential interference of the click reaction due to steric hindrance (**3**, **4**). The tridentate *N*-2-picolinic acid-aminoacetic acid-based chelator (**8**), is derived from a well characterized *N*-2-picolylamineacetic acid chelator. A similar chelator, using a pyridine instead of picolinic acid, has previously shown excellent stability and low protein binding combined with fast clearance in vivo [61]. All compounds were obtained in >90% chemical purity as determined by HPLC and their identity confirmed by NMR and mass spectroscopy (S2 File).

A common way for labeling radiotracers with ^99m^Tc is the *in situ* reduction of ^99m^TcO_4_^-^, the form in which ^99m^Tc is obtained from the ^99^Mo/^99m^Tc generator and subsequent coordination with a suitable chelator. With an overall charge of -1 and an oxidation state of +7, pertechnetate is not able to directly form complexes with chelators and is therefore often reduced using stannous chloride, a potent reducing agent. Since Tzs are susceptible to reducing conditions, this could potentially promote the formation of non-reactive dihydrotetrazines [62, 63]. The benefit of using the *fac*-[^99m^Tc(OH_2_)_3_ (CO)_3_]^+^ precursor as a synthon for radiolabeling is that the Tz is not subjected to reducing conditions that occur with the use of stannous chloride. Labeling with *fac*-[^99m^Tc(OH_2_)_3_ (CO)_3_]^+^ proceeded with high radiochemical yield (RCY) (78.3 ± 1.5%) and all Tzs were generated in high >95% radiochemical purity (RCP), determined by ITLC [64].

**Fig 3:**
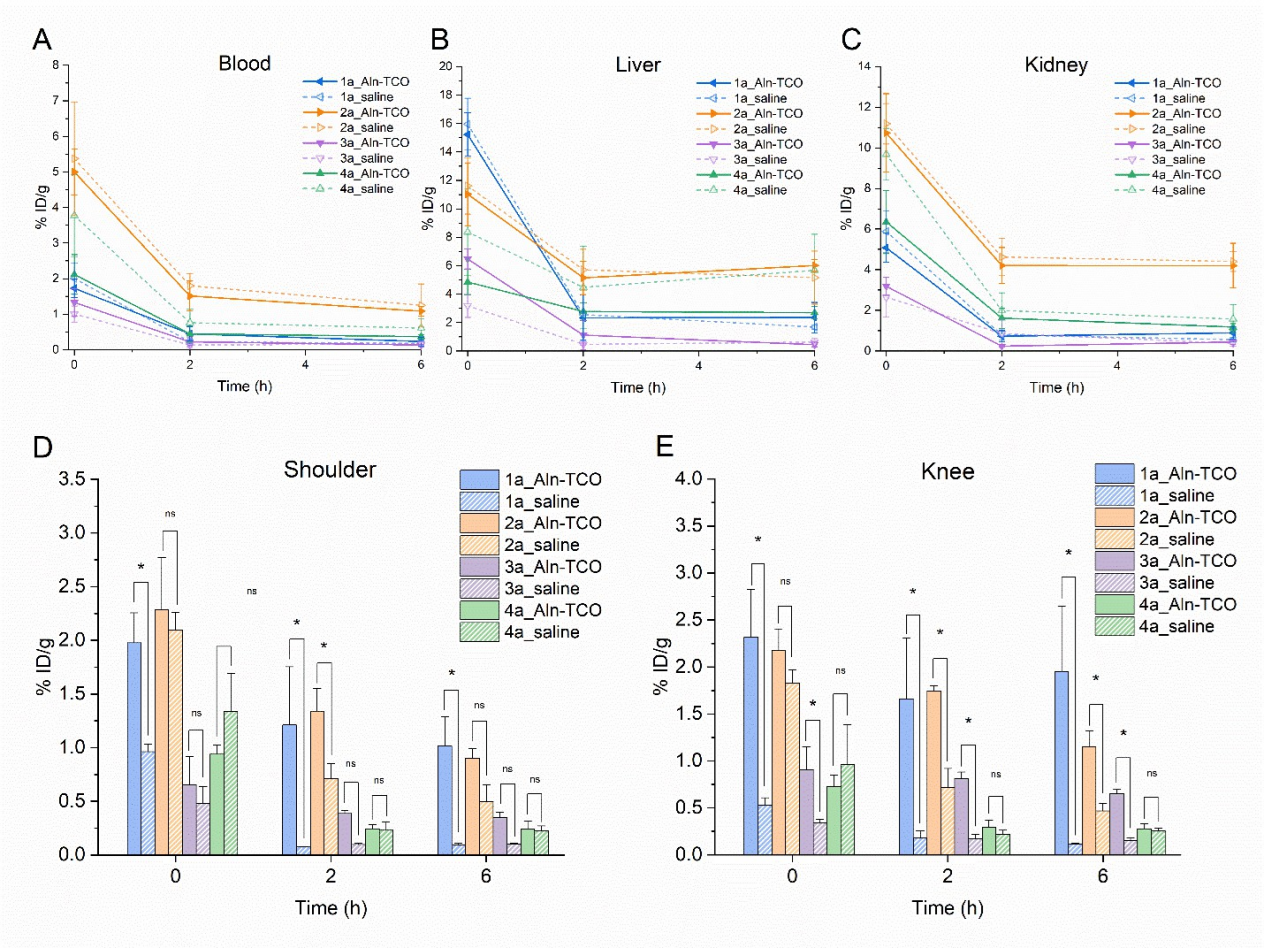
Image analysis of pretargeted bone uptake of tetrazines 1a-4a. (A-C) Organ activity in selected organs over the course of the study. All tetrazines are rapidly removed from the blood. Liver uptake decreases over time and is generally much lower than direct targeted Aln-TCO. (D) and (E) Shoulder and knee activity at 0, 2 and 6 h p.i. Tetrazines without the PEG_8_ linker show generally higher bone uptake. Differences in pretargeted tetrazines and their respective non-targeted saline controls were more pronounced in the knee. Tetrazine 4 did not show statistically significant bone uptake at any time point compared to the saline control (n = 3, mean ± SD).

To compare the performance of Tzs **1a-4a** for *in vivo* pretargeted bone uptake, a methodology similar to previous studies with Aln-TCO was applied [30, 32, 34]. Healthy mice were injected with 2 mg/kg Aln-TCO to allow accumulation of the primary pretargeting probe in knee and shoulder joints. An interval of 1 h was chosen between injection of the Aln-TCO and the ^99m^Tc-labeled Tzs. To compare the pharmacokinetics and biodistribution of the pretargeted Tzs to the pharmacokinetics of non-targeted Tzs, separate groups of mice were injected with saline prior to the administration of the Tzs. Quantitative image analysis of pretargeted vs. non-targeted Tzs showed that the pharmacokinetic profile in blood, liver and urinary system did not differ, suggesting that any residual Aln-TCO that did not bind to the bone was already cleared and no click reaction in the blood pool took place by the time Tzs were injected (Fig 3A–3C). Interestingly, the presence of the PEG_8_ linker had a strong influence on the maximum blood activity. For **1a** the maximum concentration in the blood pool amounted to 5%ID/g at time point 0 compared to 1% ID/g for Tz **3a**. Similarly, the non-PEGylated Tz **2a** showed higher blood activity compared to the same Tz scaffold with the PEG_8_ linker. Despite differences in peak blood pool activity, all Tzs were rapidly removed from circulation and little activity remains in the blood 6 h post injection.

In terms of total bone uptake, the presence of the PEG_8_ linker resulted in significantly lower uptake compared to the non-PEGylated Tzs. This effect is potentially a result of the higher activity in the blood pool of **1a** and **2a.** Limited information about the performance of Tzs using the ^99m^Tc tricarbonyl core is available. In a previous study, Bilton *et al*. labeled a series of 3-phenyl-1,2,4,5-Tzs with the ^99m^Tc tricarbonyl core and evaluated them with the Aln-TCO model. The highest bone uptake was observed for a Tz containing a PEG_5_ linker with a clogD_7.4_ of -6.78 while poor performance was observed for the same Tz with a longer PEG linker, or very lipophilic or hydrophilic Tzs [32]. In a tumor pretargeting model, Meyer *et al*. showed that higher polarity of Tz radioligands positively correlates with shorter plasma half-life [41]. Similarly, longer plasma half-life also positively correlates with click-performance. The observed difference in blood pool activity is therefore expected as PEGylated Tzs **3** and **4** have lower clogD values and hence should be excreted faster. A recent systematic study by Stéen *et al*. established that performance of *in vivo* pretargeting strongly correlated with fast kinetics of the IEDDA reaction and low lipophilicity of the Tz radioligands [42]. A potential reason why no such effect was seen in this study, is that inclusion of a PEG linker for **1** and **3** only resulted in a modest decrease in clogD. It is possible that for the tested Tzs the benefit of lower lipophilicity is outweighed by longer plasma-half life, although more data would be required to argue conclusively.

The inclusion of saline control groups offered further insight into the *in vivo* click performance of the investigated Tzs, which could be missed by only looking at total bone uptake. Even though bone uptake of the Tzs in the saline groups should only be a function of normal perfusion and tissue distribution effects and therefore minimal, image analysis shows interesting differences in the bone activity between the tested Tzs. **1a** showed statistically significant difference in bone accumulation in the pretargeted vs. non-targeted group for all time points. In contrast, bone uptake of **2a** at time point 0 was similar in the pretargeted vs. non-targeted setting and only revealed true *in vivo* pretargeting mediated differences at later time points. Due to the relative difference in joint size and overall uptake, differences in pretargeted vs. non-targeted Tzs were also more pronounced in the knee, as seen for **3a**. Notably, virtually no bone uptake was observed for **4a** in both the Aln-TCO as well as the saline group, suggesting that **4a** was unable to reach the target site (Fig 3D and 3E).

### Preparation and biodistribution of directly targeted Aln-TCO (1*)

**Fig 4:**
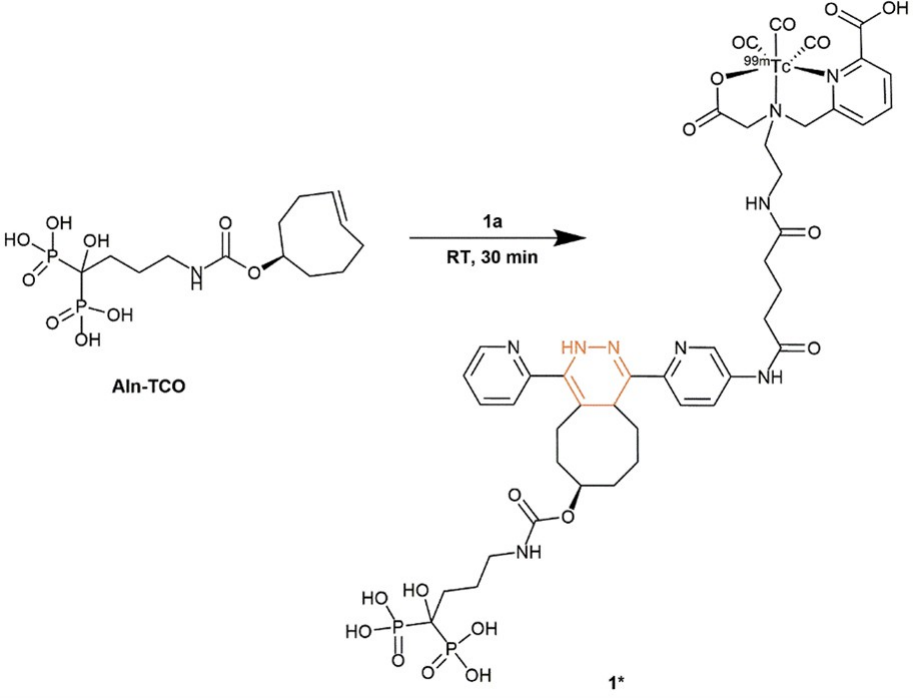
Preparation of ^99m^Tc-labeled Aln-TCO via click chemistry.

Since pretargeted bone uptake for all Tzs was relatively low, a direct targeting approach was used to determine the bone uptake of Aln-TCO as a comparison. To this end, **1a** was used as a prosthetic group to generate the bone targeting bisphosphonate (1*) (Fig 4).

**Fig 5:**
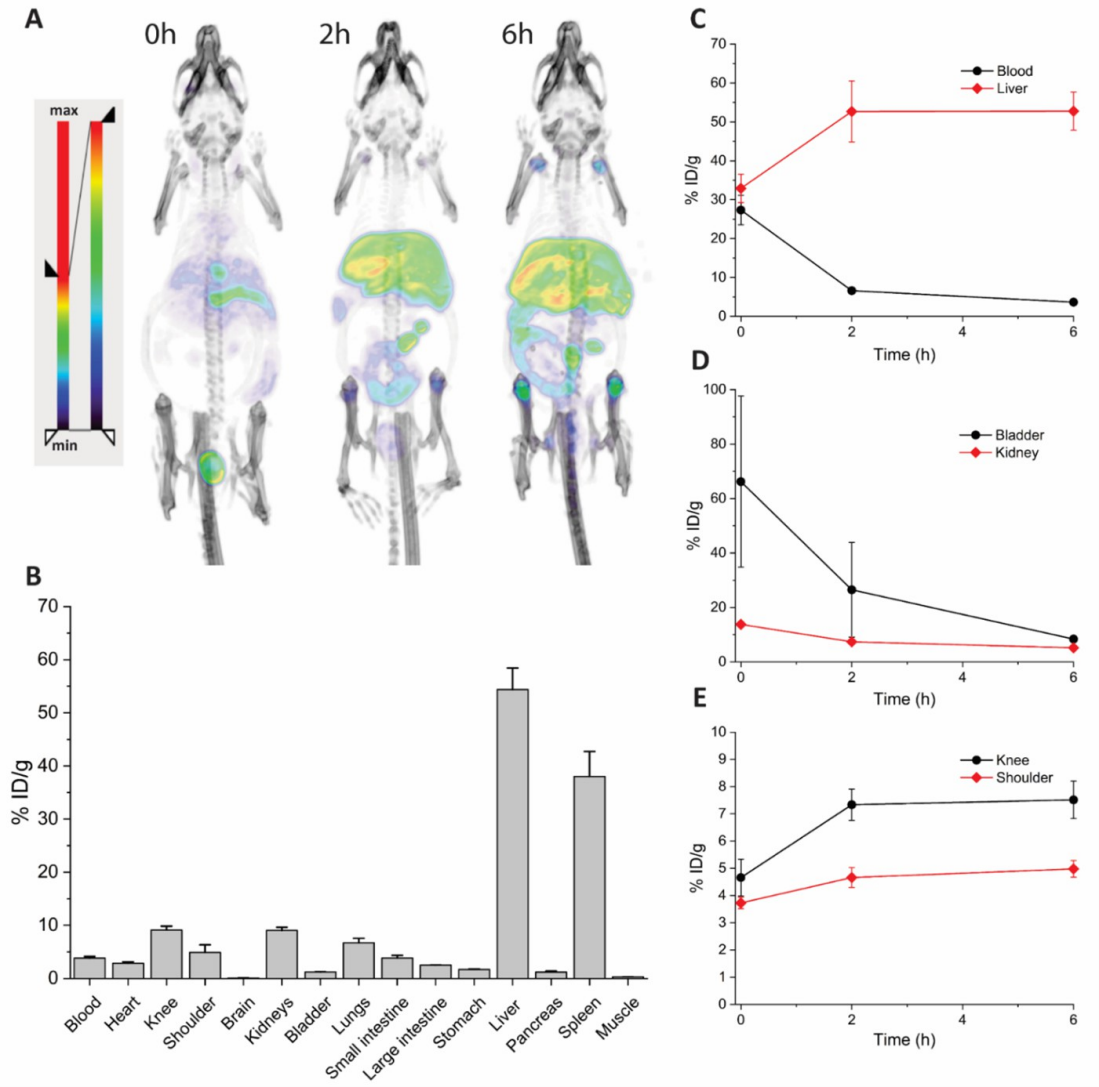
Pharmacokinetics and biodistribution of direct targeted Aln-TCO (1*). (A) Representative MIP projections of 1* in a mouse at various time points. Bone uptake in shoulder and knee is visible 2 h post injection and more pronounced after 6 h. High liver uptake indicates hepatobiliary metabolism and excretion of 1*. At 6 h p.i. noticeable activity is also found in the intestinal tract. (B) Ex vivo biodistribution of 1* 6 h p.i. High activity is found in liver and spleen, knee and shoulder uptake indicates accumumulation at sites of active bone remodeling, mostly in joint area as seen in MIP renderings. (C)-I Quantitative image analysis of various organs of interest over the course of the study. 1* is rapidly removed from the blood pool and accumulates in the liver. Urinary excretion is relatively low and high activity levels in the bladder are limited to the earliest time point. Bone uptake is visible immediately after injection and only decreases slightly over the following 6 hour period (n = 3, mean ± SD).

This compound is equivalent to the *in vivo* product of the reaction between the Aln-TCO and **1a** to enable a direct comparison between active targeting and pretargeting. Although it would be interesting to compare bone uptake of all Tzs as directly targeted IEDDA adducts, limited information would be gained, as the goal was to assess the performance of the Tzs for pretargeting. Nevertheless, the inclusion of 1* permitted a closer evaluation for at least one of the Tzs and served as a reference for the maximally achievable bone uptake of Aln-TCO in this study. It is noteworthy that contrary to previously published studies, a considerably lower dose of 2 mg/kg of Aln-TCO was used for pretargeting. Not surprisingly, higher doses of 20 mg/kg Aln-TCO in mice [30, 31] or 3 mg/kg in rats [34] resulted in higher bone uptake for other Tzs. SPECT/CT imaging of 1* in normal mice was performed analogous to the pretargeted Tzs. Notable joint uptake of 1* in knee and shoulder was visible in MIP images 6 h post injection, confirming accumulation of the bisphosphonate in areas of active bone metabolism. SPECT images further show pronounced liver uptake, suggesting hepatobiliary metabolism and excretion of 1* as the main route of elimination (Fig 5A). A post-mortem biodistribution study confirmed strong uptake of 1* in liver and spleen (Fig 5B). Knee and shoulder uptake of 9.13 ± 0.73 and 4.91 ± 1.43%ID/g, were considerably higher than pretargeted **1a** (S3 File).

Quantitative imaging revealed interesting differences between the pharmacokinetic profile of 1* and the pretargeted Tzs (Fig 5C–5E). Although the overall blood pool profile of 1* appeared similar to pretargeted Tzs, overall activity levels were drastically higher and at 0 h amounted to 27.35 ± 3.79%ID/g compared to 1.73 ± 0.26%ID/g for **1a**. At 6 h p.i., blood activity of **1a** and 1* decreased to 0.23 ± 0.13 and 3.66 ± 0.65%ID/g, respectively. This 15-fold difference in blood pool activity at 6 h p.i. clearly demonstrates drastically different pharmacokinetics between direct targeting and pretargeting. The altered pharmacokinetic profile was also reflected in liver activity, showing not only higher total values but also a different profile over time. Compared to the decreasing liver activity for the ^99m^Tc-labeled Tzs, 1* showed an increase over time. Similarly, activity in knee and shoulder increased over time, which is in agreement with the pharmacokinetic profile of alendronic acid [65]. Overall, higher bone uptake was observed for direct targeted Aln-TCO, suggesting that the tested Tzs did not react with all theoretically available TCOs at the bone.

A substantial advantage of pretargeted imaging with Tzs is the rapid excretion by virtue of their small size, which leads to better image contrast and lower off-target radiation. It is important to emphasize that it is this better contrast and site-directed accumulation, rather than a higher target uptake, which makes pretargeting such a promising technology for nuclear medicine. This is reflected in the target/background ratios that were achieved with in the pretargeted setting compared to directly targeted Aln-TCO (Table 1). In direct comparison **1a** had roughly 2-fold higher bone/blood and bone/liver ratios than 1*. Not surprisingly, better target/background ratios were reached when more time passed between injection and biodistribution. As seen for compound **3a**, where biodistributions were performed 24 h post injection, image-based activity in shoulder and knee does not appear to change over time (S4 File). However, substantially lower activity in non targeted organs was observed in the biodistribution (S3 File).

**Table 1:** Bone to organ ratios calculated from post-mortem biodistribution after last imaging timepoint (%ID/g).

	Knee/Blood	Shoulder/Blood	Knee/Liver	Shoulder/Liver
1*	2.39 ± 0.09	1.29 ± 0.30	0.17 ± 0.09	0.09 ± 0.30
**1a**	4.47 ± 0.36	2.00 ± 0.44	0.44 ± 0.29	0.20 ± 0.37
**2a**	1.63 ± 0.32	1.28 ± 0.20	0.33 ± 0.22	0.26 ± 0.10
**3a** 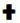	6.76 ± 0.29	3.96 ± 0.28	1.12 ± 0.18	0.66 ± 0.16
**4a**	0.71 ± 0.17	0.62 ± 0.20	0.09 ± 0.15	0.08 ± 0.21

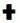
Biodistribution performed 24 h p.i., all other groups were sacrificed 6 h p.i.

## Conclusions

In conclusion we were able to evaluate four tetrazines for labeling with the ^99m^Tc tricarbonyl core with Aln-TCO as pretargeting vector. Previous structure-activity relationship studies, suggest that more hydrophilic tetrazines are able to click to the pretargeting vector better than less hydrophilic analogues [42]. In this study, two different tetrazine scaffolds were tested head-to-head with a hydrophilic PEG linker and without linker to alter their lipophilicity. To our surprise tetrazines with PEG_8_ linker showed lower bone uptake compared to their smaller counterparts or even complete lack of *in vivo* click. This suggests that apart from lipophilicity and reaction kinetics, the overall pharmacokinetic profile of the tetrazines, plays an important role in their suitability for in vivo pretargeted imaging. Of the two tested scaffolds bispyridyl substituted tetrazines performed better that the phenyl-substituted tetrazines. Despite similar total bone uptake values of the non-PEGylated Tzs **1a** and **2a**, worse target/background ratios and higher passive bone accumulation of **2a** suggests a better *in vivo* performance of the bispyridyl substituted tetrazine. This is corroborated by the fact that **4a** did not click at all. Our results suggest that **1a** showed the best pharmacokinetic profile and could serve as the basis for the development of other ^99m^Tc-labeled Tzs.

## Supporting information

S1 File Synthesis of Tz-precursors. (DOCX)

S2 File Characterization of ligands. (DOCX)

S3 File Biodistribution data. (DOCX)

S4 File Quantitative image analysis of compound 3a. (DOCX)

S5 File (PDF)

S6 File (PDF)

S7 File (PDF)

S8 File (PDF)

## Abbreviations

ALN: alendronate
ESI: electron spray ionization
IEDDA: inverse electron-demand Diels-Alder
ITLC: instant thin layer chromatography
PET: positron emission tomography
RT: room temperature
SPECT: single-photon emission computed tomography
TCO: trans-cyclooctene
TZ: tetrazine
VOI: volume of interest
