# Supplementary material for: Development of a 99mTc-labeled tetrazine for pretargeted SPECT imaging using an alendronic acid-based bone targeting model
Source: PLoS One. 2024 Apr 16;19(4):e0300466. doi: 10.1371/journal.pone.0300466 (PMC11020896; doi:10.1371/journal.pone.0300466)
Supplement: S2 File — (DOCX) [file pone.0300466.s002.docx]

## Characterization of ligands

Purity and identity of ligands 1-4 were assessed on an ACQUITY UPLC H-Class chromatography system (Waters, Milford, MA) coupled online to a photodiode array detector (absorbance recorded at 254 nm, Waters) and an electrospray ionization source on a mass spectrometer (QDa detector, Waters). Separation was achieved on a Waters Acquity BEH-C18 column (inner diameter: 1.1 mm; length: 50 mm; particle size: 1.7 μm, Waters) at a flow rate of 0.3 mL/min. The mobile phase consisted of solvent A (0.1% v/v formic acid (FA) in water) and solvent B (0.1% v/v FA in 90% aqueous acetonitrile), running in a linear gradient from 5%B to 95%B in 12 to 15 minutes. Note that for purity analysis of (**3**) a Waters Alliance e2695 separations module coupled to a Waters 2489 UV/VIS detector as described in the radiochemistry section.

### 6-(((carboxymethyl)(2-(5-oxo-5-((6-(6-(pyridin-2-yl)-1,2,4,5-tetrazin-3-yl)pyridin-3-yl)amino)pentanamido)ethyl)amino)methyl)picolinic acid (**1**)

Calculated mass 600.22, found 601.05 [M+H]^+^

### 6-(((2-(5-((4-(1,2,4,5-tetrazin-3-yl)phenyl)amino)-5-oxopentanamido)ethyl)(carboxymethyl)amino)methyl)picolinic acid (**2**)

Calculated mass 522.20, found 522.91 [M+H]^+^

### 6-(2-(carboxymethyl)-6,34,38-trioxo-38-((6-(6-(pyridin-2-yl)-1,2,4,5-tetrazin-3-yl)pyridin-3-yl)amino)-9,12,15,18,21,24,27,30-octaoxa-2,5,33-triazaoctatriacontyl)picolinic acid (**3**)

Calculated mass: 1023.47, found 1024.27 [M+H]^+^

### 6-(38-((4-(1,2,4,5-tetrazin-3-yl)phenyl)amino)-2-(carboxymethyl)-6,34,38-trioxo-9,12,15,18,21,24,27,30-octaoxa-2,5,33-triazaoctatriacontyl)picolinic acid (**4**)

Calculated mass: 945.44, found 946.29 [M+H]^+^
