# Supplementary material for: Development of a 99mTc-labeled tetrazine for pretargeted SPECT imaging using an alendronic acid-based bone targeting model
Source: PLoS One. 2024 Apr 16;19(4):e0300466. doi: 10.1371/journal.pone.0300466 (PMC11020896; doi:10.1371/journal.pone.0300466)
Supplement: S3 File — (DOCX) [file pone.0300466.s003.docx]

## Biodistribution Data

**S3 Table:** Post-mortem biodistribution of Tzs **1a-4a** and **1*** in % ID/g of tissue

|  | **1a pretargeted** | **1a non-targeted** | **2a pretargeted** | **2a non-targeted** | **3a pretargetedᶧ** | **3a non-targetedᶧ** | **4a pretargeted** | **4a non-targeted** | **1*** |
| --- | --- | --- | --- | --- | --- | --- | --- | --- | --- |
| **Blood** | 0.29 ± 0.12 | 0.33 ± 0.27 | 1.11 ± 0.37 | 1.96 ± 0.34 | 0.09 ± 0.03 | 0.09 ± 0.08 | 0.36 ± 0.03 | 0.99 ± 0.63 | 3.82 ± 0.32 |
| **Heart** | 0.19 ± 0.18 | 0.11 ± 0.05 | 0.55 ± 0.06 | 0.63 ± 0.17 | 0.03 ± 0.00 | 0.05 ± 0.04 | 0.14 ± .02 | 0.38 ± 0.23 | 2.87 ± 0.27 |
| **Knee** | 1.29 ± 0.25 | 0.04 ± 0.01 | 1.81 ± 0.38 | 0.58 ± 0.08 | 0.58 ± 0.10 | 0.05 ± 0.05 | 0.26 ± 0.03 | 0.25 ± 0.13 | 9.13 ± 0.73 |
| **Shoulder** | 0.58 ± 0.16 | 0.04 ± 0.00 | 1.42 ± 0.13 | 0.46 ± 0.04 | 0.34 ± 0.05 | 0.04 ± 0.05 | 0.22 ± 0.04 | 0.25 ± 0.15 | 4.91 ± 1.43 |
| **Brain** | 0.02 ± 0.01 | 0.01 ± 0.01 | 0.04 ± 0.01 | 0.04 ± 0.00 | 0.00 ± 0.00 | 0.02 ± 0.03 | 0.01 ± 0.00 | 0.03 ± 0.02 | 0.10 ± 0.01 |
| **Kidney** | 0.94 ± 0.20 | 0.86 ± 0.08 | 3.36 ± 3.74 | 8.59 ± 0.59 | 0.46 ± 0.06 | 0.77 ± 0.50 | 2.76 ± 0.37 | 3.74 ± 1.07 | 9.04 ± 0.60 |
| **Bladder** | 0.21 ± 0.07 | 0.17 ± 0.13 | 0.99 ± 0.09 | 0.77 ± 0.36 | 0.07 ± 0.03 | 0.22 ± 0.25 | 0.40 ± 0.15 | 0.56 ± 0.21 | 1.22 ± 0.04 |
| **Lung** | 0.29 ± 0.05 | 0.29 ± 0.12 | 1.67 ± 0.43 | 1.59 ± 0.36 | 0.08 ± 0.01 | 0.15 ± 0.14 | 0.32 ± 0.03 | 0.73 ± 0.47 | 6.70 ± 0.85 |
| **Intestine** | 3.62 ± 1.08 | 2.21 ± 0.37 | 1.49 ± 0.14 | 1.91 ± 1.18 | 0.07 ± 0.04 | 0.07 ± 0.06 | 0.45 ± 0.14 | 0.64 ± 0.37 | 3.85 ± 0.49 |
| **Stomach** | 0.41 ± 0.03 | 0.59 ± 0.30 | 0.99 ± 0.22 | 0.85 ± 0.20 | 0.20 ± 0.27 | 0.11 ± 0.11 | 0.28 ± 0.06 | 0.40 ± 0.18 | 1.70 ± 0.10 |
| **Liver** | 2.96 ± 0.94 | 2.09 ± 0.30 | 5.44 ± 0.51 | 4.60 ± 0.28 | 0.52 ± 0.04 | 0.98 ± 0.74 | 2.83 ± 0.36 | 2.28 ± 0.34 | 54.39 ± 4.03 |
| **Pancreas** | 1.16 ± 0.56 | 1.54 ± 0.97 | 0.74 ± 0.34 | 0.68 ± 0.26 | 0.04 ± 0.01 | 0.05 ± 0.05 | 0.14 ± 0.02 | 0.25 ± 0.12 | 1.20 ± 0.20 |
| **Spleen** | 0.23 ± 0.11 | 0.25 ± 0.17 | 0.96 ± 0.15 | 0.72 ± 0.07 | 0.08 ± 0.01 | 0.10 ± 0.10 | 0.21 ± 0.03 | 0.43 ± 0.19 | 38.03 ± 4.69 |
| **Muscle** | 0.07 ± 0.03 | 0.07 ± 0.04 | 0.17 ± 0.02 | 0.13 ± 0.03 | 0.02 ± 0.00 | 0.01 ± 0.00 | 0.05 ± 0.01 | 0.11 ± 0.05 | 0.31 ± 0.01 |

ᶧsacrificed after 24 h, all other animals sacrificed after 6 h imaging time point.
