# Supplementary material for: Development of a 99mTc-labeled tetrazine for pretargeted SPECT imaging using an alendronic acid-based bone targeting model
Source: PLoS One. 2024 Apr 16;19(4):e0300466. doi: 10.1371/journal.pone.0300466 (PMC11020896; doi:10.1371/journal.pone.0300466)
Supplement: S5 File — (PDF) [file pone.0300466.s005.pdf]

S1 File. Synthesis of Tz-precursors

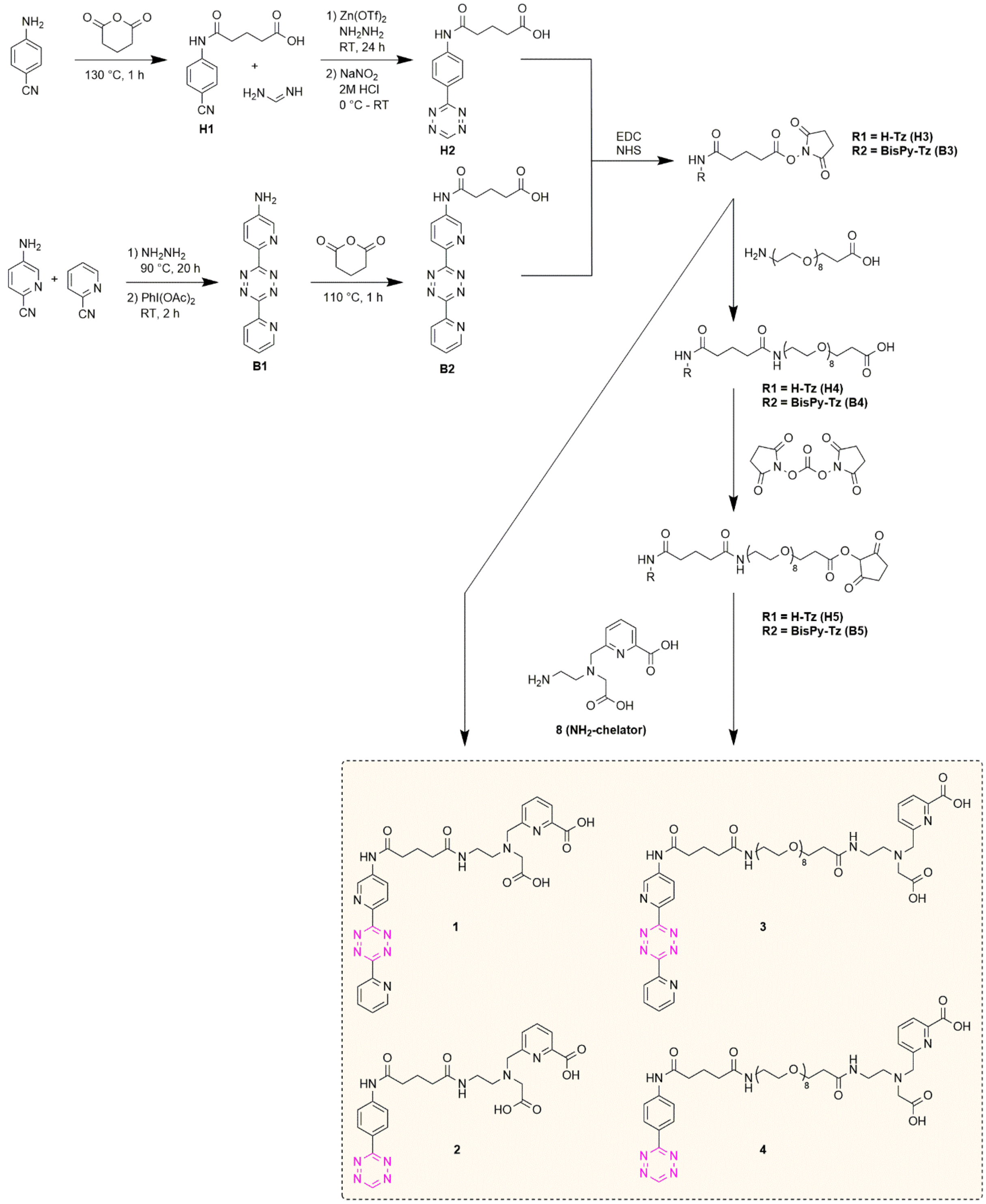

S1 Scheme 1. *Synthesis approach for tetrazines 1-4.*

*5-((4-Cyanophenyl)amino)-5-oxopentanoic acid (**H1**)*

4-Aminobenzonitrile (1 g, 8.5 mmol) and glutaric anhydride (4.849 g, 42.5 mmol) were added to a dried microwave vial. The vial was capped and 15 mL of dry THF was added. After heating the suspension for

1 h at 130 °C in a microwave and cooling to room temperature, the crude product precipitated from the mixture. THF was removed under reduced pressure and the crude product was recrystallized from methanol two times to afford the product as an off-white solid (1.299 g, 65% yield). ^{1}H NMR: (400 MHz, DMSO-d6): δ 12.08 (s, 1H); 10.33 (s, 1H); 7.80-7.73 (m, 4H); 2.41 (t, J = 7.4 Hz, 2H); 2.29 (t, J = 7.3 Hz, 2H); 1.82 (p, J = 7.4 Hz, 2H) ppm.^{13}C NMR: (400 MHz, DMSO-d6): δ 174.5; 172.1; 143.9; 133.7; 119.5; 105.1; 36.0; 33.3; 20.6 ppm.

*5-((4-(1,2,4,5-Tetrazin-3-yl)phenyl)amino)-5-oxopentanoic acid (**H2**)*

H1 (250 mg, 1.08 mmol), formamidine acetate (1.118 g, 10.75 mmol and zinc trifluoromethanesulfonate (105 mg, 0.54 mmol) were suspended in 4.3 mL ethanol in a microwave vial. The vial was capped, hydrazine monohydrate (3.9 mL, 80.40 mmol) was added, and the solution was stirred at room temperature for 48 h. The reaction mixture was cooled to 0 °C and diluted with water before adding sodium nitrite (734 mg, 10.63 mmol). 2M HCl was added slowly until the solution turned bright pink and a pH of 3 was reached. At this point evolution of gas ceased and the solution was allowed to warm up to room temperature. The solution was extracted 3 times with EtOAc. The organic phase was washed with brine, dried over sodium sulfate and evaporated to yield the crude product as a bright pink solid. The crude product was adsorbed onto celite and purified by dry column vacuum chromatography [63] on C-18 modified silica using a gradient of 95:5 to 20:80 H_2O:MeOH in fractions of 20 mL each to yield the title compound as a bright pink solid (43 mg, 13% yield). ^{1}H NMR: (600 MHz, DMSO-d6): δ 12.51 (s, 1H); 10.36 (s, 1H); 8.46 (d, 2H), 7.90 (d, 2H); 2.44 (t, 2H); 2.31 (t, 2H); 1.84 (p, 2H) ppm.

*2,5-Dioxopyrrolidin-1-yl 5-((4-(1,2,4,5-tetrazin-3-yl)phenyl)amino)-5-oxopentanoate (**H3**)*

H2 (39 mg, 0.14 mmol), N-hydroxysuccinimide (NHS) (50 mg, 0.43 mmol) and 1-ethyl-3-(3-dimethylaminopropyl)carbodiimide (EDC) (70 mg, 0.37 mmol) were added to a dried microwave vial. Dry DMF (1 mL) was added and the solution was stirred for 18 h at room temperature. Water was added to the reaction mix until the product precipitated. The suspension was centrifuged (14000 RCF, 10 min) and the supernatant was decanted. The pink product was subjected to two more cycles of

resuspension and centrifugation to yield the title compound as a pink solid (25 mg, 46% yield). ^{1}H NMR: (400 MHz, DMSO-d6): δ 10.52 (s, 1H); 10.38 (s, 1H); 8.47 (s, 2H); 7.90 (s, 2H); 2.83 (s, 4H); 2.79 (t, 2H); 2.54 (t, 2H); 1.97 (p, 2H) ppm. ^{13}C NMR: (400 MHz, DMSO-d6): δ 171.4; 170.7; 169.2; 165.6; 158.2; 143.9; 129.2; 126.4; 119.8; 35.2; 30.1; 25.9; 20.4 ppm.

*6-(6-(Pyridin-2-yl)-1,2,4,5-tetrazin-3-yl)pyridin-3-amine (**B1**)*

2-cyanopyridine (0.9 g, 8.64 mmol), 5-amino-2-cyanopyridine (1.03 g, 8.64 mmol) and hydrazine monohydrate (64-65 % in water, 3.9 ml, 80 mmol) where successively added to a microwave vial at room temperature. The vial was sealed and the mixture heated to 90°C for 18 h under stirring. The resulting orange solid was transferred into a separating funnel and extracted with EtOAc to remove residual hydrazine. The organic phase was dried over $MgSO_4$, filtered and the solvent was evaporated to obtain 1.13 g of an orange solid. The solid was dissolved in dichloromethane, adsorbed onto celite and purified by column chromatography using a gradient elution (EtOAc-heptane 3:10 to pure EtOAc). The eluted fractions containing the title compound were combined and the solvent was removed to yield 6-(6-(pyridin-2-yl)-1,4-dihydro-1,2,4,5-tetrazin-3-yl)pyridin-3-amine (278mg, 13%). ^{1}H NMR (400 MHz, DMSO-d6): δ 8.69 (s, 1H), 8.65 (s, 1H), 8.60–8.64 (m, 1H), 7.87–7.98 (m, 3H), 7.64 (d, J = 8.6, 1H), 7.51 (ddd, J = 7.2 and J = 4.9 and J = 1.5, 1H), 6.99 (dd, J = 8.6 and J = 2.7, 1H), 5.87 (s, 2H) ppm.

6-(6-(Pyridin-2-yl)-1,4-dihydro-1,2,4,5-tetrazin-3-yl)pyridin-3-amine (268 mg, 1.01 mmol) was oxidized by adding (diacetoxyiodo)benzene (390 mg, 1.21 mmol) in one portion in 50 mL DCM. The solution was stirred at room temperature for 2 h. The solvent was removed under reduced pressure the crude product was redissolved in DCM and adsorbed onto celite. The crude material was purified by column chromatography using a gradient elution (DCM–MeOH, 100:1 to 100:10) to afford pure product as a dark red solid (152 mg, 60%). ^{1}H NMR (600 MHz, DMSO-d6) δ 8.90 (dd, J = 4.4, 2.2 Hz, 1H), 8.56 – 8.51 (m, 1H), 8.37 (dd, J = 8.5, 1.4 Hz, 1H), 8.24 (t, J = 2.0 Hz, 1H), 8.18 – 8.10 (m, 1H), 7.75 – 7.67 (m, 1H), 7.13 (ddd, J = 8.7, 2.9, 1.5 Hz, 1H), 6.36 (s, 2H) ppm.

*5-Oxo-5-((6-(6-(pyridin-2-yl)-1,2,4,5-tetrazin-3-yl)pyridin-3-yl)amino)pentanoic acid (**B2**)*

B1 (100 mg, 0.39 mmol) and Glutaric anhydride (227 mg, 1.97 mmol) were added to a microwave vial. The vial was capped and 10 mL dry THF was added. The vial was heated to 110°C for 1 h and cooled to room temperature. The crude product was filtered over a fritted glass filter and washed with acetone, EtOAc, DCM and Et2O (50 mL each) to afford the product as a pink solid (75 mg, 52%). ^{1}H NMR (600 MHz, DMSO-d6) δ 12.10 (s, 1H), 10.56 (s, 1H), 9.05 (d, J = 2.6 Hz, 1H), 8.94 (d, J = 4.6 Hz, 1H), 8.65 – 8.57 (m, 2H), 8.43 (dt, J = 8.7, 2.7 Hz, 1H), 8.19 – 8.11 (m, 1H), 7.75 – 7.68 (m, 1H), 2.57 (m, 2H), 2.36 – 2.30 (m, 2H), 1.87 (h, J = 9.2, 7.5 Hz, 2H) ppm.

*2,5-Dioxopyrrolidin-1-yl 5-oxo-5-((6-(6-(pyridin-2-yl)-1,2,4,5-tetrazin-3-yl)pyridin-3-yl)amino)pentanoate (**B3**)*

B2 (483 mg, 1.32 mmol), N-(3-dimethylaminopropyl)-N′-ethylcarbodiimide hydrochloride (340 mg, 1.78 mmol) and N-hydroxysuccinimide (212 mg, 1.85 mmol) were added to a dried flask which was evacuated and backfilled with nitrogen three times. 6 mL anhydrous DMF was added and the mixture was stirred at room temperature for 24 h. The progress of the reaction was monitored via TLC (8% MeOH in DCM, Rf bispyridine tetrazine-NHS: 0.48). After 24 h 102 mg (0.59 mmol) EDC and 63 mg (0.62 mmol) NHS were added and the mixture was stirred for 12 more hours until the TLC indicated full conversion of the starting compound. DMF was removed under reduced pressure and the crude product was resuspended in 10 mL DCM and transferred to a falcon tube. The suspension was centrifuged for 10 minutes at 14000 RCF and the supernatant was decanted. This process was repeated 2 times with DCM and 2 times with MeOH. The product was dried under vacuum to yield the product as a purple flakey solid (432 mg, 82%). ^{1}H NMR (600 MHz, DMSO-d6) δ 10.62 (s, 1H), 9.06 (d, J = 2.5 Hz, 1H), 8.94 (dd, J = 4.7, 1.6 Hz, 1H), 8.63 (d, J = 8.6 Hz, 1H), 8.60 (d, J = 7.8 Hz, 1H), 8.44 (dd, J = 8.8, 2.6 Hz, 1H), 8.16 (tt, J = 7.6, 1.4 Hz, 1H), 7.73 (dd, J = 7.6, 4.7 Hz, 1H), 2.83 – 2.79 (m, 6H), 2.59 (t, J = 7.4 Hz, 2H), 1.99 (p, J = 7.4 Hz, 2H) ppm. ^{13}C NMR (151 MHz, DMSO-d6) δ 171.96, 170.72, 169.26, 163.53, 151.07, 150.68, 144.36, 141.80, 138.89, 138.27, 127.04, 126.69, 125.36, 124.66, 35.10, 32.79, 30.03, 25.94, 20.25 ppm.

Synthesis of chelator

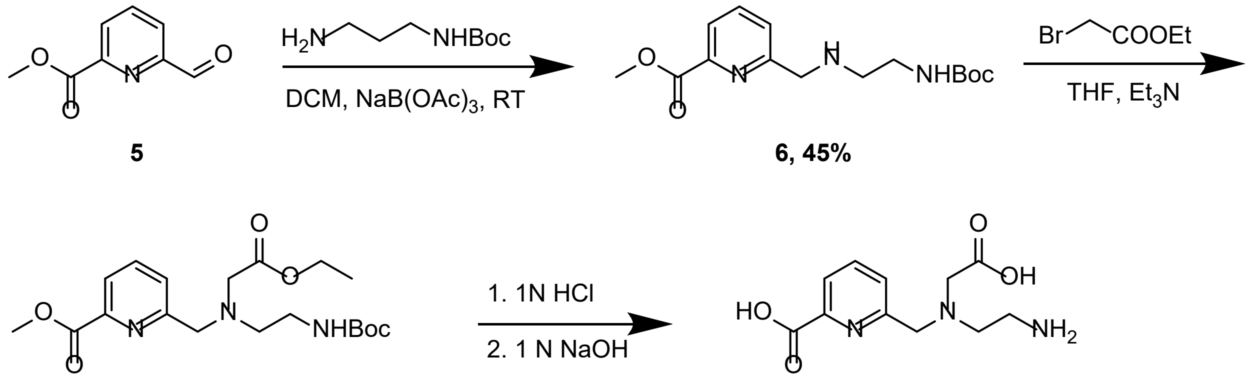

5 — 6, 45% — 7, 90% — NH2-chelator (8)

S1 Scheme 2: *Synthesis of amine functionalized chelator*

Synthesis of the amine functionalized chelator involves reductive amination of the starting aldehyde **5** with *N*-boc-ethylenediamine and sodium triacetoxyborohydride in anhydrous DCM to give the secondary amine **6** in 74% yield. This is transformed into compound **7** in 90% by alkylation with ethyl 2-bromoacetate and Et_3N in anhydrous THF. The next steps involve boc-deprotection and hydrolysis of the ester functions to afford the target compound.

Methyl 6-(((2-((tert-butoxycarbonyl)amino)ethyl)amino) methyl) picolinate (**6**)

To a solution of **5** (233 mg, 1.4 mmol) and *N*-boc-ethylenediamine (0.22 mL) in anhydrous DCM (5.8 mL) was added $NaB(OAc)_3$ (418 mg) under N_2 and the reaction mixture was stirred at room temperature for 3 h. The resulting mixture was diluted with DCM (6 mL) and sat. $NaHCO_3$ (6 mL) was added. The organic layer was separated, and the aqueous layer was extracted with DCM (3 x 6 mL). The combined organic extracts were dried over Na_2SO_4, and the solvent was removed under reduced pressure. The residue was purified by FCC (DCM/MeOH 5%) to afford pure **2** (205 mg, 47%) along with 188 mg early fractions [12-14] subject to further purification. The second purification was performed using a less polar eluent system (DCM/MeOH 4%) to afford an additional amount of **2** (120 mg) for a total yield of 325 mg, (74%)

^{1}H NMR (400MHz, $CDCl_3$): δ 7.84 (d, 1H, J7.6Hz), 7.64 (t, 1H, J15.5, 7.1Hz), 7.41 (d, 1H, J7.7Hz), 5.31 (br s, 1H), 3.86 (s, 2H), 3.82 (s, 3H), 3.10 (br t, 2H), 2.65 (t, 2H, J10.8, 5.6Hz), 1.25 (s, 9H) ppm. ^{13}C

NMR (106MHz, $CDCl_3$): 165.58, 159.99, 156.05, 147.31, 137.41, 125.67, 123.51, 78.85, 54.32, 52.75, 48.73, 40.05, 28.30 ppm.

Methyl 6-(((2-((tert-butoxycarbonyl)amino)ethyl)(2-ethoxy-2-oxoethyl)amino)methyl)picolinate (**7**)

A mixture of amine **6** (196mg, 0.64mmol), Et_3N (0.33 mL) and ethyl 2-bromoacete (0.3 mL) in anhydrous THF (12.5 mL) was refluxed for 4 h. After cooling to room temperature, the solvent was evaporated under reduced pressure to give crude **3** that was purified by FCC on silica gel using DCM/MeOH 2% as an eluent to provide pure compound **3** (170 mg, 68%). ^{1}H NMR (400 MHz, $CDCl_3$): δ 7.98 (dd, 1H, J 7.4, 1.0Hz), 7.79 (t, 1H, J 15.6, 7.6Hz),7.70 (d, 1H, J8.0Hz), 5.25 (br s, 1H),3.99 (br s 2H), 3.95 (br s, 3H), 3.57 (s, 1H), 3.15 (m, 2H), 2.76 (t, 2H, J12.1, 5.3Hz), 1.36 (s, 9H), 1.22 (t, 3H, J14.5, 7.2Hz) ppm. ^{13}C NMR (106MHz, $CDCl_3$): δ 171.66, 165.93, 160.33, 156.26, 147.533, 137.72, 126.31, 123.95, 60.88, 60.41, 55.66, 54.33, 38.69, 28.61, 28.51, 14.37 ppm.

6-(((2-Aminoethyl)(carboxymethyl) amino)methyl) picolinic acid (**8**)

Step 1. A mixture of compound **7** (184 mg, 0.46mmol) in 1N HCl (6 mL) was refluxed for 24 h. Then the H_2O was removed under reduced pressure to give a solid (106 mg) which was used for the next step without purification. **Step 2**. To the solid from step 1 (106 mg) 1N NaOH (5 mL) was added, and the mixture was refluxed for 5 h and then acidified with 1N HCl until pH 6 was attained. The H_2O was removed under reduced pressure and the solid obtained was treated with MeOH (10 mL), filtered and dried *in vacuo* to afford compound **8** (151 mg). ^{1}H NMR (400 MHz, $CDCl_3$): δ 7.98 (br d, 1H, J8.0 Hz), 7.89 (br d, 1H, J 7.3Hz), 7.60 (br d, 2H, J 7.3Hz), 4.10 (br s, 2H), 3.39 (br d, 2H), 3.10 (br s, 2H), 3.04 (br s, 1H).
