# Supplementary material for: Development of a 99mTc-labeled tetrazine for pretargeted SPECT imaging using an alendronic acid-based bone targeting model
Source: PLoS One. 2024 Apr 16;19(4):e0300466. doi: 10.1371/journal.pone.0300466 (PMC11020896; doi:10.1371/journal.pone.0300466)
Supplement: S7 File — (PDF) [file pone.0300466.s007.pdf]

S3 File. Biodistribution Data

S3 Table: Post-mortem biodistribution of Tzs **1a-4a** and **1*** in % ID/g of tissue

	1a pretargeted	1a non-targeted	2a pretargeted	2a non-targeted	3a pretargeted[ǂ]	3a non-targeted[ǂ]	4a pretargeted	4a non-targeted	1*
**Blood**	0.29 ± 0.12	0.33 ± 0.27	1.11 ± 0.37	1.96 ± 0.34	0.09 ± 0.03	0.09 ± 0.08	0.36 ± 0.03	0.99 ± 0.63	3.82 ± 0.32
**Heart**	0.19 ± 0.18	0.11 ± 0.05	0.55 ± 0.06	0.63 ± 0.17	0.03 ± 0.00	0.05 ± 0.04	0.14 ± .02	0.38 ± 0.23	2.87 ± 0.27
**Knee**	1.29 ± 0.25	0.04 ± 0.01	1.81 ± 0.38	0.58 ± 0.08	0.58 ± 0.10	0.05 ± 0.05	0.26 ± 0.03	0.25 ± 0.13	9.13 ± 0.73
**Shoulder**	0.58 ± 0.16	0.04 ± 0.00	1.42 ± 0.13	0.46 ± 0.04	0.34 ± 0.05	0.04 ± 0.05	0.22 ± 0.04	0.25 ± 0.15	4.91 ± 1.43
**Brain**	0.02 ± 0.01	0.01 ± 0.01	0.04 ± 0.01	0.04 ± 0.00	0.00 ± 0.00	0.02 ± 0.03	0.01 ± 0.00	0.03 ± 0.02	0.10 ± 0.01
**Kidney**	0.94 ± 0.20	0.86 ± 0.08	3.36 ± 3.74	8.59 ± 0.59	0.46 ± 0.06	0.77 ± 0.50	2.76 ± 0.37	3.74 ± 1.07	9.04 ± 0.60
**Bladder**	0.21 ± 0.07	0.17 ± 0.13	0.99 ± 0.09	0.77 ± 0.36	0.07 ± 0.03	0.22 ± 0.25	0.40 ± 0.15	0.56 ± 0.21	1.22 ± 0.04
**Lung**	0.29 ± 0.05	0.29 ± 0.12	1.67 ± 0.43	1.59 ± 0.36	0.08 ± 0.01	0.15 ± 0.14	0.32 ± 0.03	0.73 ± 0.47	6.70 ± 0.85
**Intestine**	3.62 ± 1.08	2.21 ± 0.37	1.49 ± 0.14	1.91 ± 1.18	0.07 ± 0.04	0.07 ± 0.06	0.45 ± 0.14	0.64 ± 0.37	3.85 ± 0.49
**Stomach**	0.41 ± 0.03	0.59 ± 0.30	0.99 ± 0.22	0.85 ± 0.20	0.20 ± 0.27	0.11 ± 0.11	0.28 ± 0.06	0.40 ± 0.18	1.70 ± 0.10
**Liver**	2.96 ± 0.94	2.09 ± 0.30	5.44 ± 0.51	4.60 ± 0.28	0.52 ± 0.04	0.98 ± 0.74	2.83 ± 0.36	2.28 ± 0.34	54.39 ± 4.03
**Pancreas**	1.16 ± 0.56	1.54 ± 0.97	0.74 ± 0.34	0.68 ± 0.26	0.04 ± 0.01	0.05 ± 0.05	0.14 ± 0.02	0.25 ± 0.12	1.20 ± 0.20
**Spleen**	0.23 ± 0.11	0.25 ± 0.17	0.96 ± 0.15	0.72 ± 0.07	0.08 ± 0.01	0.10 ± 0.10	0.21 ± 0.03	0.43 ± 0.19	38.03 ± 4.69
**Muscle**	0.07 ± 0.03	0.07 ± 0.04	0.17 ± 0.02	0.13 ± 0.03	0.02 ± 0.00	0.01 ± 0.00	0.05 ± 0.01	0.11 ± 0.05	0.31 ± 0.01

[ǂ]sacrificed after 24 h, all other animals sacrificed after 6 h imaging time point.
