# Supplementary material for: Development of a 99mTc-labeled tetrazine for pretargeted SPECT imaging using an alendronic acid-based bone targeting model
Source: PLoS One. 2024 Apr 16;19(4):e0300466. doi: 10.1371/journal.pone.0300466 (PMC11020896; doi:10.1371/journal.pone.0300466)
Supplement: S8 File — (PDF) [file pone.0300466.s008.pdf]

S4 File. Quantitative Image Analysis of ^{99m}Tc Labeled Compound 3a

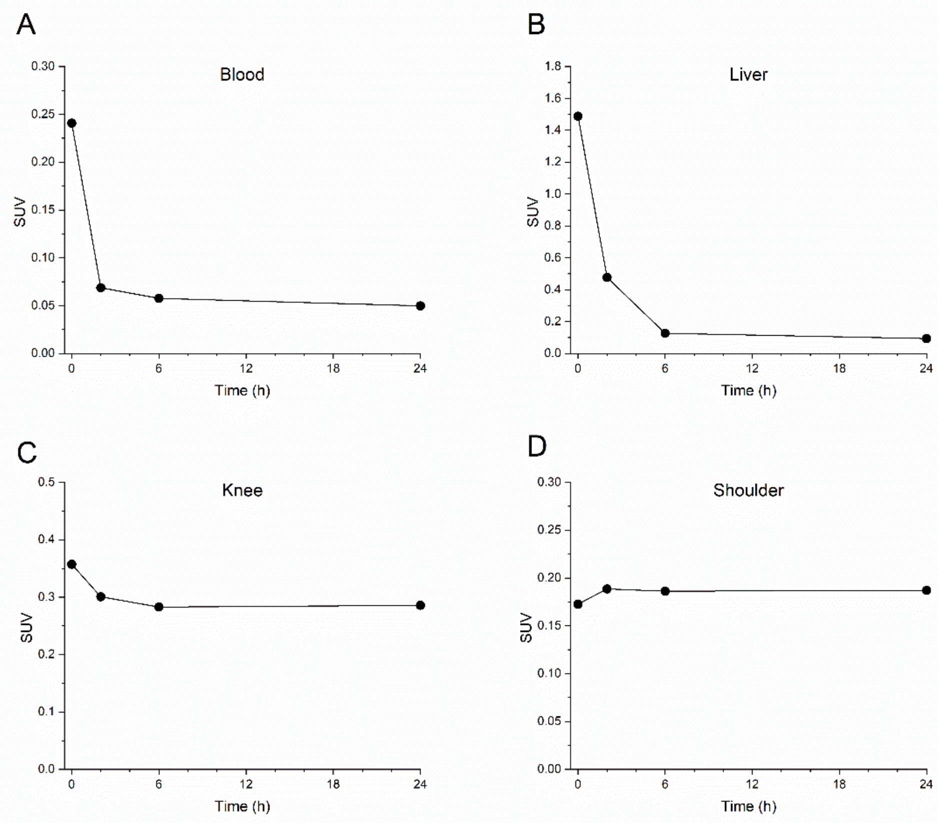

***S4 Figure: Quantitative image analysis of 3a**. Imaging at 24 h was only performed for 1 animal. Blood (A) and liver (B) show fast excretion following injection. Activity in the Knee (C) and shoulder (D) joint remain constant for extended periods of time, suggesting stable incorporation of the Tz at the bone surface.*
